# Neuromechanistic causes of timber falls in older adults with mild cognitive impairment: Is response initiation or motor execution the problem?

**DOI:** 10.1007/s11357-025-01910-4

**Published:** 2025-10-24

**Authors:** Jessica Pitts, Shuaijie Wang, Tanvi Bhatt

**Affiliations:** Department of Physical Therapy, University of Illinois at Chicago, 1919 W Taylor St, Chicago, IL 60612 USA

**Keywords:** Mild cognitive impairment, Reactive balance, Fall risk, Alzheimer’s Disease, Reactive stepping, Muscle synergies

## Abstract

Older adults with mild cognitive impairment (OAwMCI) show reactive balance deficits compared to cognitively intact older adults (CIOA), which could increase the likelihood of ‘Timber’ falls (i.e., backward falls caused by extremely delayed reactive stepping). This study investigated potential neuromechanistic causes of Timber falls in OAwMCI, including delayed muscle onset latencies and/or altered muscle synergies during reactive stepping. 36 OAwMCI, 38 CIOA, and 20 young adults were exposed to a large anterior stance perturbation, with electromyography collected from the biceps femoris (BF), vastus lateralis (VL), medial gastrocnemius (MG), and tibialis anterior (TA). Timber falls were identified by falling (> 30% weight in harness) without initiating stepping within 430 ms (perturbation duration). Timber falls only occurred in OAwMCI, who were subcategorized into MCI: Timber (36%) or MCI: Step (intact stepping, 64%). MCI: Timber had higher fall rate, lower reactive stability, shorter step length, and longer step initiation time compared to groups with intact stepping (*p* < *0.05*), and delayed onsets of stepping limb muscles and the stance limb MG (*p* ≤ *0.03*). MCI: Timber also showed many structural differences in muscle synergies (M1-6), such as recruiting a unique synergy (M4) which might affect coordination of the stepping limb MG, and failing to recruit M6, which might be involved in taking a long recovery step (stepping limb VL). These results suggest that reactive balance deficits in OAwMCI may be related to problems with both initiating and executing reactive stepping, possibly due to neural pathology and sensorimotor processing deficits which delay response initiation and/or affect motor command execution.

## Introduction

Fall incidence is 2× higher in older adults with mild cognitive impairment (OAwMCI) than in cognitively intact older adults (CIOA) [1, 2], which can lead to severe health-related consequences (e.g., injury, sedentary behavior) [3, 4]. Heightened fall risk in OAwMCI could stem from deficits within the reactive balance control system, which is responsible for restoring stability of the center of mass (COM) relative to the base of support (BOS) when unexpected perturbations (e.g., slips, trips) occur [5, 6], often via reactive stepping [7, 8]. When exposed to slip-like perturbations, our lab has shown that OAwMCI have higher fall rate, lower reactive center of mass (COM) stability, and altered reactive stepping kinematics compared to CIOA [9–11]. However, these studies focused exclusively on kinematic outcomes, and the underlying neuromechanistic causes of reactive balance deficits in OAwMCI remain unknown.

Reactive balance deficits in OAwMCI could be driven by issues with 1) response initiation (*which could be reflected by delayed lower limb muscle onset latencies*) and/or 2) response execution (*which could be reflected by altered muscle synergy patterns during reactive stepping*). However, it is unclear whether both of these aspects are affected by MCI pathology, or if one aspect contributes more substantially to deterioration of the reactive balance response. Reactive lower limb muscle activity is proposed to be triggered via brainstem loops once a perturbation is detected [12, 13], which could be affected in OAwMCI due to impaired sensorimotor processing/integration [14–16] and/or neural damage in the brainstem (e.g., reduced grey matter volume) [17–19]. OAwMCI have shown delayed muscle onset latencies compared to CIOA when initiating volitional movements [20, 21], although it is unknown if similar results occur in unpredictable contexts. Further, once the response is initiated, complex interlimb neuromuscular coordination is needed to execute a stability-restoring reactive step while simultaneously generating sufficient net extensor torque to prevent limb collapse on the standing limb [22, 23]. Muscle synergy analysis has been used by many studies to investigate multi-muscle activation patterns during perturbation exposure [24–28], and muscle synergies are proposed to be recruited via higher cortical centers and combined to produce complex movements [29–31]. Studies have shown that aging can subtly affect both structural and temporal components of muscle synergies during reactive balance tasks [24, 32], and it is possible that MCI pathology could further affect the selection and/or execution of these motor commands due to neural damage in motor areas (e.g., motor cortex, brainstem, cerebellum) [33, 34]. However, studies have not yet compared muscle synergies between CIOA and OAwMCI during reactive balance tasks.

Pathological changes to the initiation and/or execution of reactive balance responses in OAwMCI may not only affect fall outcome (i.e., fall vs. recovery), but also affect the type of fall which occurs. For example, falls can be caused by ineffective stepping which does not generate sufficient joint torques to restore stability, or can be caused by delayed or absent stepping which results in the COM state moving outside of a recoverable range before a response is initiated [5, 35]. The Postural Stress Test identified one specific type of backwards fall, known as the “Timber” response, which occurs because no corrective responses are initiated to restore stability after a perturbation occurs [36, 37]. However, after being identified on the Postural Stress Test over three decades ago, Timber falls have been majorly overlooked or ignored in the literature, with studies mainly investigating differences in biomechanical/neuromuscular strategies between fallers and non-fallers, without sub-categorizing Timber falls. Understanding the neuromechanisms underlying these Timber falls could help develop tailored strategies for their prevention, especially given the risk of severe impact-related injuries when reactive stepping is delayed or absent (e.g., hip fracture, head injury) [38, 39]. Further, while conducting our previous study [11], we observed that OAwMCI demonstrated Timber falls more often than CIOA, although we did not formally analyze group differences in their occurrence.

The purpose of this study was to investigate potential neuromechanistic causes of reactive balance impairments and Timber falls in OAwMCI, including deficits in response initiation (i.e., delayed lower limb muscle onset latencies) and/or deficits in response execution (i.e., altered muscle synergy patterns). During exposure to a support surface perturbation, we hypothesized that OAwMCI with Timber falls would have delayed lower limb muscle onset latencies, potentially caused by sensorimotor processing deficits which interfere with perturbation detection and response triggering (*i.e., deficits in response initiation*). Further, we hypothesized that OAwMCI with Timber falls would recruit less effective muscle synergies for balance recovery (different structures), potentially caused by damage to neural centers involved in the selection and/or execution of motor commands (*i.e., deficits in response execution*). These neuromuscular changes in OAwMCI could be reflected by different reactive stepping kinematics (delayed step initiation, shorter step length), which are less effective for restoring reactive COM stability. The results of this study will provide an in-depth understanding of how cognitive impairment affects the intricate dynamics of reactive balance control and risk of potentially injurious Timber falls. Further, investigating neuromuscular deficits in OAwMCI will provide potential training targets for future fall prevention interventions in this population.

## Methods

### Participants

Before completing any outcome assessments, all participants first completed a screening assessment to determine their eligibility for the study. Potential participants were only included if they did not report any acute or chronic neurological, musculoskeletal, or systemic diagnosis, had not had major surgery in the past 6 months, were not hospitalized in the past 3 months, were able to walk for at least 10 m without an assistive device, and were not taking any Alzheimer’s Disease or dementia modifying medications. Additionally, all older adults were screened for osteoporosis on a heel bone density scan (Lunar Achilles Tendon (EXPII)) and excluded if they received a T-score < −2.0. In total, 74 older adults (ages 55–90) and 20 young adults (ages 18–35) passed the screening assessment and were included in the study. This study was approved by the Institutional Review Board at the University of Illinois at Chicago (2021–0478; 2022–0136) and all participants provide informed consent.

The Montreal Cognitive Assessment (MoCA) was used to classify older adults as either OAwMCI (score 18–25, n = 36) or CIOA (score ≥ 26, n = 38) [40]. Participants who scored < 18 were excluded, as this score indicates dementia. MCI classification was further confirmed using the Jak/Bondi criteria, which included a battery of tests within domains of memory (Wechsler Memory Scale subtests for auditory, visual, immediate, and delayed memory [41]), language (Category Fluency Test [42], Boston Naming Test [43]), and processing speed/executive function (Trail Making Tests A and B [44]). Participants were identified as OAwMCI if they scored greater than one standard deviation below the normative means (obtained via published literature [42, 45, 46]) on at least two or more measures within at least one domain. The sample size in older adult groups (CIOA, OAwMCI) was higher than the sample size of YA to account for higher variability of electromyography (EMG) data with aging [47–49]. Further, the purpose of including YA data was to distinguish MCI-related effects from age-related effects.

### Experimental protocol

Participants were exposed to a forward support surface translation while standing on a motorized ActiveStep treadmill (Simbex, NH, USA). Once the trial began, the perturbation was delivered at a random time within 10 s. Participants were warned before the trial that a perturbation would be delivered, although they were not aware of when the perturbation would occur. A high perturbation intensity was selected to ensure that reactive stepping would be required to successfully prevent a fall (displacement: 33 cm; acceleration: 21.75 m/s^2^). Participants wore a full-body safety harness connected to an overhead I-bar during the perturbation test to prevent any contact with the ground other than the feet.

### Data collection and analysis

#### Kinematic data

Kinematic data were collected with a sampling frequency of 120 Hz from an 8-camera motion capture system (Qualisys Motion Capture, Goteberg, Sweden). A Helen Hayes marker set was used to place 26 reflective markers on the participant and one marker was placed on the treadmill for reference. Individual marker trajectories were gap-filled and low-pass filtered using a fourth order Butterworth filter (cutoff: 6 Hz). A custom MATLAB code (MathWorks, MA, USA) was used to calculate kinematic outcomes of reactive balance performance.

**Fall Rate**: We classified the outcome of each trial as a fall or a recovery. A fall was identified if the load cell connected to the harness was weighted by greater than 30% body weight, and visually verified using video recordings [50].

**Margin of Stability (MOS)**: The margin of stability (MOS) was calculated using Equation 1 [51], and used to quantify the stability of the COM state (position and velocity) relative to the base of support (BOS):

1 $$MOS={COM}_{x}+\frac{{COM}_{v}}{\sqrt{\frac{g}{l}} }-\mathrm{BOSx}$$

COMx and COMv indicate the COM position and velocity relative to the BOS in the anterior-posterior direction, ‘g’ is the gravitational constant, ‘l’ is the leg length, and BOSx is the posterior border (i.e., heel) of the most posterior foot which is in contact with the ground. MOS was calculated at the instant of 0.4 seconds after perturbation onset, to ensure that the movement of the treadmill belt was equivalent for all trials [10]. MOS was normalized by foot distance (i.e., distance between the stepping foot heel and the standing foot toe). Negative MOS values indicate that the COM is not stable relative to the BOS.

**Recovery Step Length:** Recovery step length was calculated as the difference between the right and left heel markers at recovery step touchdown.

**Step Initiation Time:** Step initiation time was calculated as the time difference between the onset of the perturbation and liftoff of the recovery stepping foot (foot completely cleared from ground). All time events were visually identified using marker trajectories in Qualisys software.

**Step Execution Time:** Step execution time was calculated as the time difference between the liftoff of recovery stepping and the touchdown of recovery stepping.

#### Identification of Timber falls

**Fig. 1:**
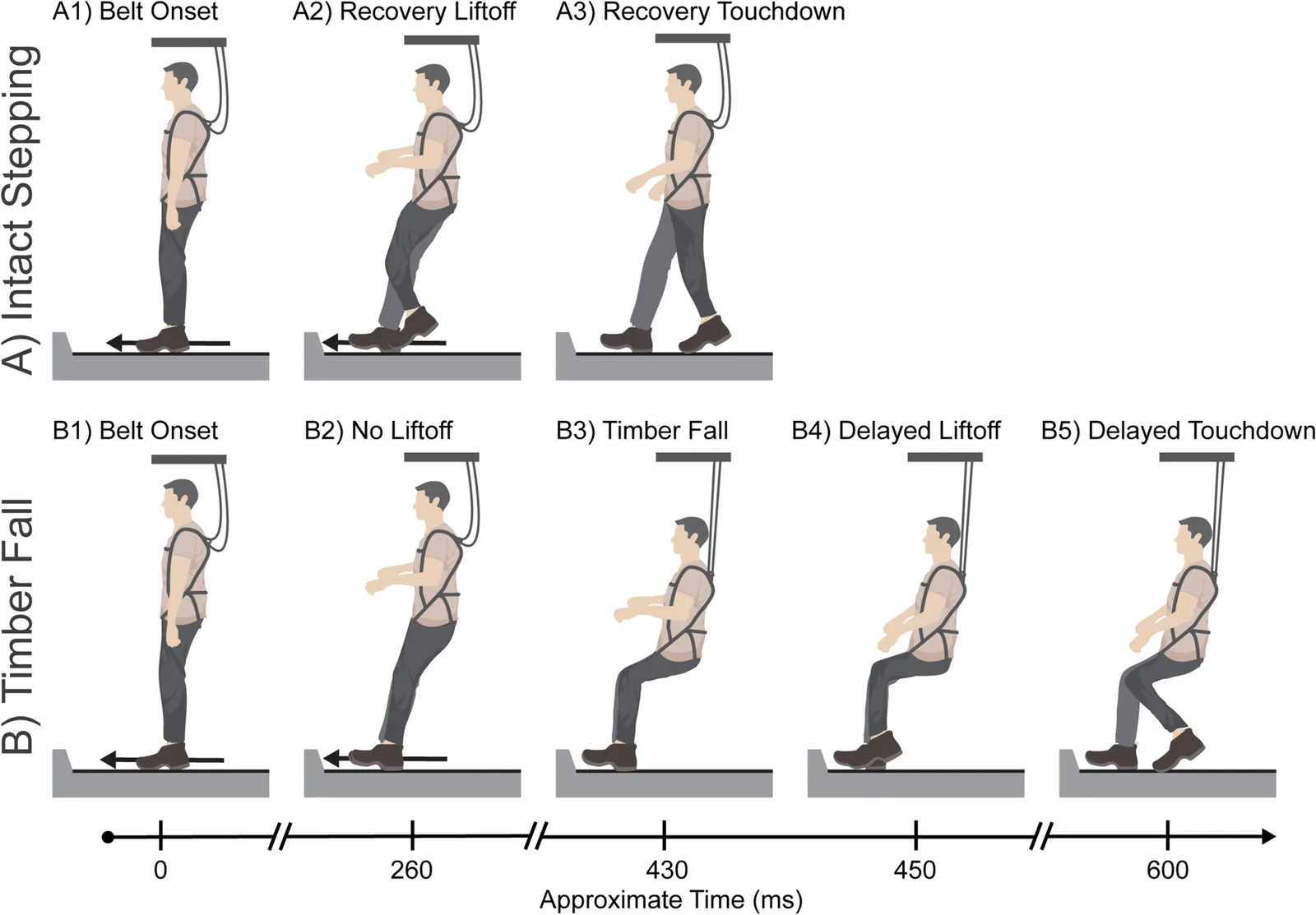
Schematic example of an intact reactive stepping response (top row, A) compared to a Timber fall (bottom row, B). Perturbation onset occurred when the treadmill belt began to accelerate in the forward direction (A1, B1). In an intact stepping response, a reactive step was initiated before the belt stopped moving (A2), followed by recovery touchdown (A3). Reactive stepping did not always prevent falling, although a no fall outcome is pictured here for demonstration purposes. Timber falls were identified if participants failed to initiate a recovery step while the perturbation was ongoing (B2) and fell backwards into the harness (loaded by > 30% body weight) (B3). Participants with Timber falls showed delayed step initiation (B4) and completion (B5) only after the perturbation had ended. The timeline across the bottom represents estimated event times, based on mean liftoff and touchdown times for CIOA and MCI: Step (for intact stepping) and MCI: Timber (for Timber falls)

Participants were categorized as having a Timber fall if they 1) fell into the harness (> 30% body weight identified via load cell) and 2) did not initiate a recovery step before the perturbation was completed (i.e., treadmill belt finished acceleration and steady state velocity phases, 430 ms after belt onset). A schematic representation of the time course of a Timber fall compared to an intact reactive stepping response can be found in Fig. 1.

13 out of 36 OAwMCI (36%) had Timber falls and were subcategorized as MCI: Timber. The other 23 out of 36 OAwMCI (64%) had intact stepping responses and were subcategorized as MCI: Step. None of the included CIOA or YA demonstrated Timber falls and were not subcategorized. Thus, four groups were included in the analysis (i.e., YA, CIOA, MCI: Step, MCI: Timber), and all participants in the dataset were included in analysis of both kinematic and neuromuscular outcomes. Participant demographics and cognitive performance scores for each group can be found in Table 1.

**Table 1:** Participant demographic information in young adults (YA), cognitively intact older adults (CIOA), older adults with mild cognitive impairment with intact stepping responses (MCI: Step), and older adults with mild cognitive impairment with Timber falls (MCI: Timber), presented as mean ± SD. Cognitive performance on the Montreal Cognitive Assessment (MoCA) and Jak/Bondi assessment battery are also shown for older adult groups. The last column indicates the main effect of group (p-value) on each outcome, using either one-way ANOVA (continuous variables) or Chi-square test (binomial variables). Significant group effects (p<0.05) are indicated by a bolded p-value

	YA	CIOA	MCI: Step	MCI: Timber	p-value
Age (years)	24.9 ± 3.2	68.6 ± 6.8	64.7 ± 6.4	69.0 ± 6.2	** < 0.001**
Sex (F/M)	7/13	16/22	6/17	3/10	0.484
Height (m)	1.7 ± 0.1	1.7 ± 0.1	1.7 ± 0.2	1.7 ± 0.2	0.522
Weight (kg)	67.7 ± 13.7	78.7 ± 14.9	78.1 ± 15.3	81.1 ± 19.0	**0.039**
MoCA (/30)	N/A	27.0 ± 2.3	22.3 ± 2.6	22.8 ± 2.3	** < 0.001**
Jak/Bondi Tests					
*Memory Domain*					
WMS – Auditory Index	N/A	113 ± 14	89 ± 9	87 ± 10	** < 0.001**
WMS – Visual Index	N/A	111 ± 13	88 ± 17	89 ± 17	** < 0.001**
WMS – Immediate Index	N/A	114 ± 15	87 ± 10	86 ± 11	** < 0.001**
WMS – Delayed Index	N/A	112 ± 14	85 ± 11	90 ± 11	** < 0.001**
*Language Domain*					
Category Fluency (#)	N/A	47.9 ± 11.2	34.1 ± 9.2	35.6 ± 6.9	** < 0.001**
Boston Naming (/60)	N/A	52.9 ± 6.4	45.4 ± 6.5	46.5 ± 9.0	**0.001**
*Executive Function/Processing Domain*					
Trail Making A (s)	N/A	32.4 ± 10.1	51.6 ± 19.6	50.8 ± 19.1	** < 0.001**
Trail Making B (s)	N/A	66.2 ± 22.4	134.1 ± 74.3	150.6 ± 76.3	** < 0.001**

*MoCA: Montreal Cognitive Assessment; WMS: Wechsler Memory Scale*

#### Neuromuscular data

Wireless surface EMG was collected bilaterally from four lower limb muscles, including the biceps femoris (BF), vastus lateralis (VL), medial gastrocnemius (MG), and tibialis anterior (TA) at a sampling frequency of 600 Hz (Delsys Inc., MA, USA). EMG data recordings were time-synchronized with marker data.

**Fig. 2:**
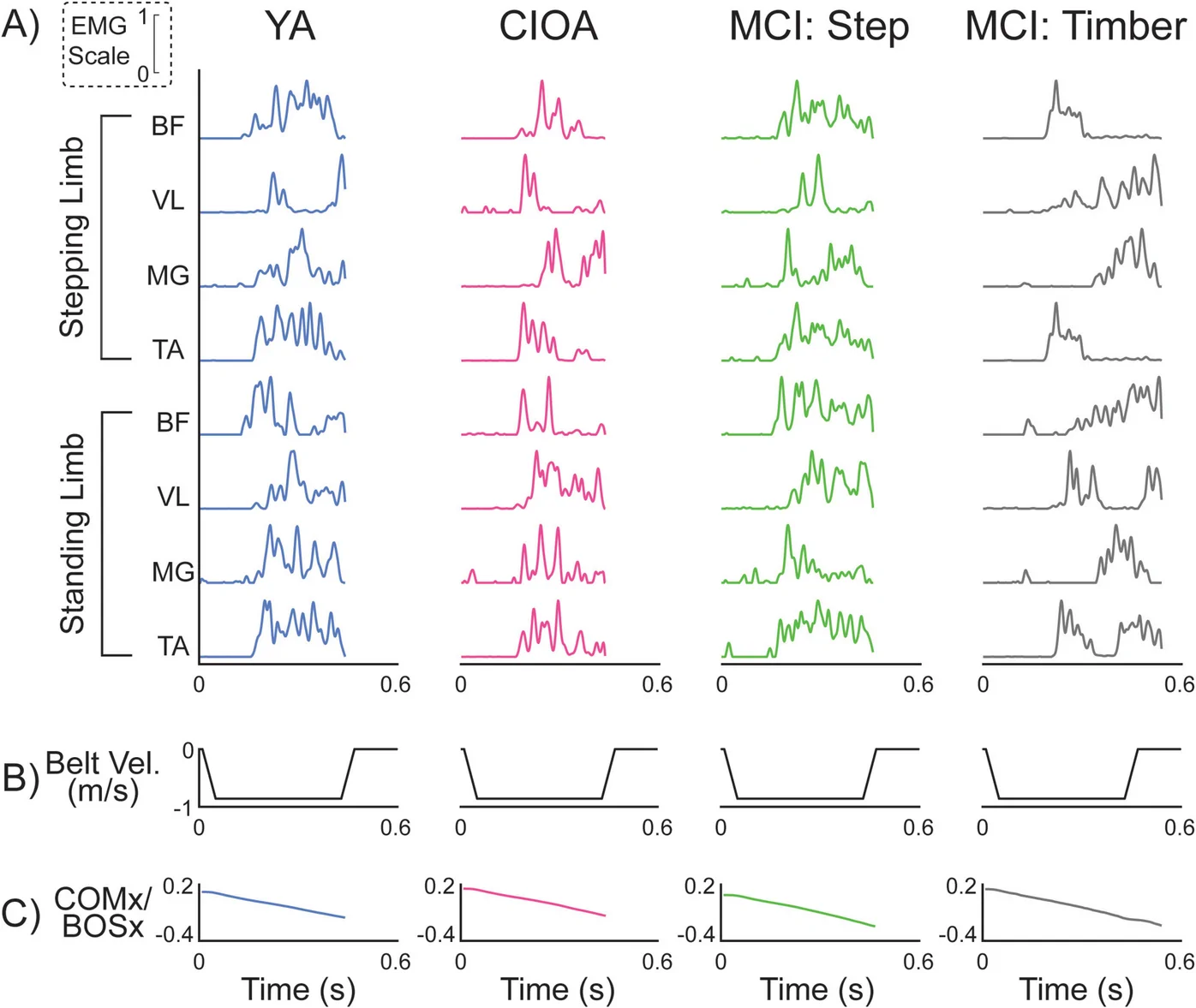
Representative tracings of filtered electromyography (EMG) data during unexpected perturbation exposure for one young adult (YA), cognitively intact older adult (CIOA), older adult with mild cognitive impairment with an intact stepping response (MCI: Step), and older adult with mild cognitive impairment with a Timber fall (MCI: Timber) (A). EMG data were recorded from the bilateral biceps femoris (BF), vastus lateralis (VL), medial gastrocnemius (MG), and tibialis anterior (TA), and are plotted from 10 ms before perturbation onset to 10 ms after recovery step touchdown. Also shown are the corresponding onset and intensity of the perturbation (treadmill belt velocity, B), and position of the center of mass relative to the base of support (here the standing heel) (COMx/BOSx) for each individual (C). Negative treadmill belt velocity indicates that the belt is moving in an anterior direction and causing the feet to move forward, and more negative COMx/BOSx values indicate that the position of the COM is posterior to the standing heel

Before beginning data analysis, EMG signals were filtered using a second-order, high-pass filter with a cutoff of 35 Hz, and the mean EMG signal was subtracted from all data points to center baseline activation at 0 for each channel. EMG signals were then full-wave rectified, and low-pass filtered with a cutoff of 40 Hz. EMG data for each trial was trimmed from 10 ms before perturbation onset until 10 ms after recovery step touchdown, and all channels were normalized to the maximum activation in this range. This method of EMG normalization has been used by previous studies investigating lower limb neuromuscular control during perturbation exposure [24, 52, 53]. Marker data and video recordings were used to identify which limb was used for recovery stepping in each trial, and muscles were subsequently identified as being on the recovery stepping limb or standing (i.e., non-stepping) limb. A representative tracing of filtered EMG data for each group can be found in Fig. 2.

**Onset Latencies**: Onset latencies for each muscle were determined as the time between perturbation onset and the time at which the EMG activity exceeded two standard deviations of the baseline for at least 30 ms (i.e., pre-perturbation) activity [27, 54]. Onset latencies were first auto-detected using a custom MATLAB code, and then manually verified and adjusted if needed.

**Muscle Synergies**: To extract muscle synergies, filtered EMG data were first down-sampled by averaging the EMG data in 15 ms time bins [24, 55], then all channels were temporarily scaled to have unit standard deviation = 1 so that each muscle would be equally weighted. Channels were reverted to their original form after synergy extraction. Muscle synergies were extracted using non-negative matrix factorization (NNMF), which assumes that each muscle activation pattern (M) consists of multiple muscle synergies with time-invariant spatial structures (W_*i*_), each activated by a temporal coefficient (c_*i*_) [27, 56]. Thus, a specific muscle activation pattern (M) could be characterized by Equation 2.

2 $$\mathrm{M}={\mathrm{c}}_{1}{\mathrm{cW}}_{1}+ {\mathrm{c}}_{2}{\mathrm{W}}_{2}+ \dots + {\mathrm{c}}_{n}{\mathrm{W}}_{n}$$

The number of synergies for each participant was determined as the lowest number of synergies needed to adequately reconstruct the EMG response, which was considered as >75% variance accounted for (VAF) in each muscle and >90% VAF overall [24, 29]. Within each group (YA, CIOA, MCI: Step, MCI: Timber), extracted synergies were pooled across participants and then grouped using a hierarchical cluster analysis [24, 27]. Clustered synergies were ordered by their time of peak recruitment (averaged across all groups) following perturbation onset.

### Statistical analysis

Demographics (age, height, weight, MoCA, Jak/Bondi tests) were compared between groups using one-way ANOVAs, and sex was compared between groups using a Chi-square test. Kinematic outcomes (reactive COM stability, step initiation time, step execution time, recovery step length) were compared between groups using one-way ANOVAs, and fall rate was compared between groups using a Chi-square test, where fall outcome was coded as a binomial variable (1: fall; 0: recovery).

Onset latencies for each individual muscle and the number of recruited muscle synergies were compared between groups using one-way ANOVAs. The structural similarity of muscle synergies between groups was examined by calculating Pearson correlation coefficients between the average structural vectors (W_i_) from each clustered synergy. A pair of synergies were considered similar if r was > 0.834, based on the critical value which represents statistical similarity (*p* = *0.01*) for 8 muscles [24, 27]. We also examined the contribution of each muscle to each synergy (ranging from 0 to 1), and considered a muscle to be an important contributor if the mean contribution was ≥ 0.4 [24]. All ANOVAs were followed up with post-hoc pairwise comparisons with a Bonferroni correction, and the alpha level was set at 0.05.

## Results

### Demographics

There was a significant main effect of group on age (F(3,90) = 264.636, *p* < *0.001*) and weight (F(3,90) = 2.913, *p* = *0.039*). Age was lower in YA compared to CIOA, MCI: Step, and MCI: Timber (*p* < *0.001*), although not significantly different between any older adult groups (*p* ≥ *0.092*). Weight was not significantly different between any groups after accounting for Bonferroni corrections. There was no significant main effect of group on sex (X^2^(3) = 2.453, *p* = *0.484*) or height (F(3,90) = 0.756, *p* = *0.522*).

There was a significant main effect of group on MoCA (F(2,71) = 32.460, *p* < *0.001*) and all Jak/Bondi assessments, including WMS – Auditory (F(2,71) = 25.660, *p < 0.001*), WMS – Visual (F(2,71) = 16.271, *p < 0.001*), WMS – Immediate (F(2,71) = 28.961, *p < 0.001*), WMS – Delayed (F(2,71) = 27.217, *p < 0.001*), Category Fluency (F(2,71) = 11.496, *p < 0.001*), Boston Naming (F(2,71) = 7.469, *p = 0.001*), Trail Making A (F(2,71) = 11.791, *p < 0.001*), and Trail Making B (F(2,71) = 15.438, *p < 0.001*). For the MoCA and all Jak/Bondi assessments, cognitive performance was lower in both MCI: Step and MCI: Timber compared to CIOA (*p* < *0.05*), although there were no significant differences between MCI: Step and MCI: Timber (*p* > *0.05*).

### Kinematic outcomes

**Fig. 3:**
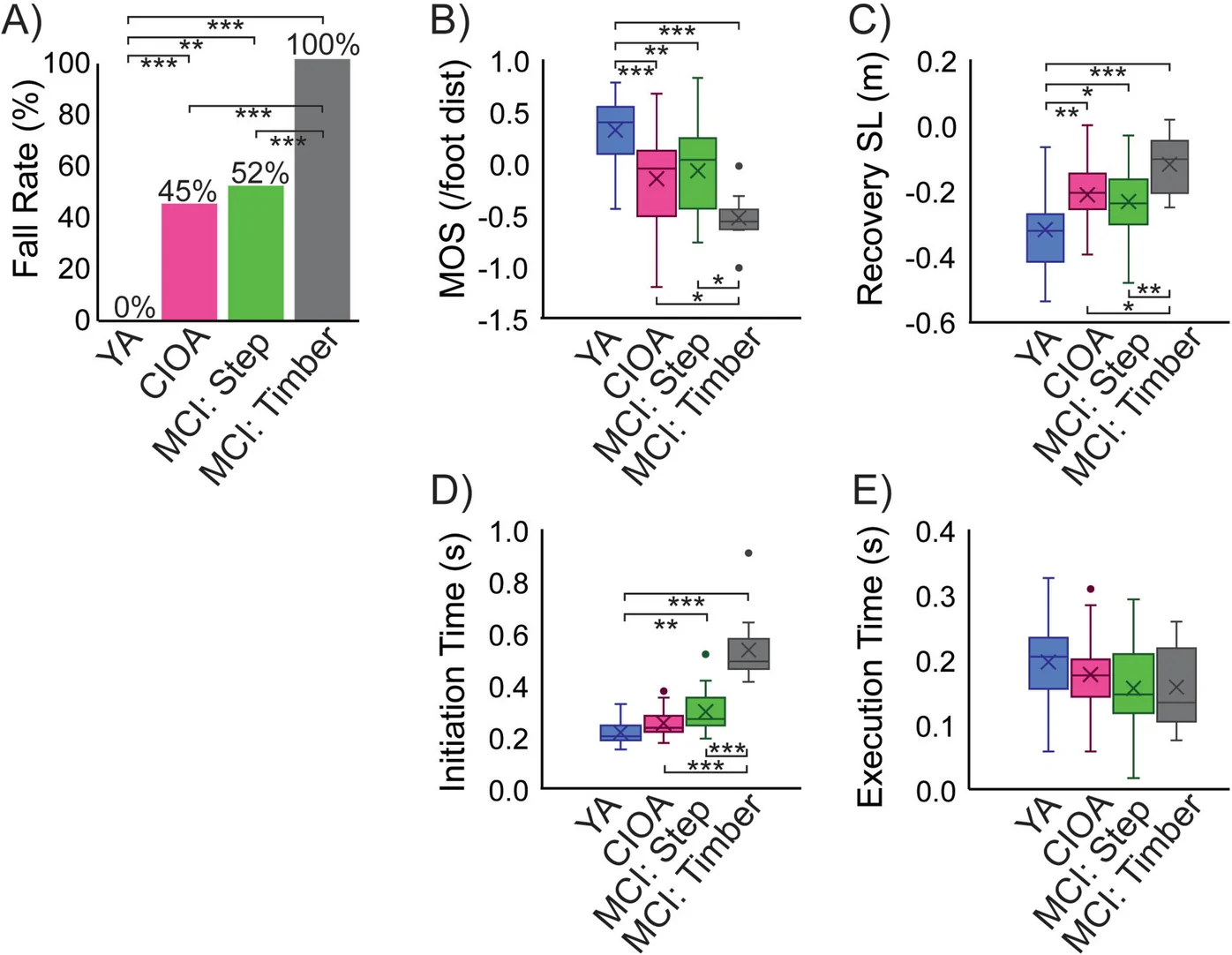
Kinematic outcomes during unexpected perturbation exposure in young adults (YA), cognitively intact older adults (CIOA), older adults with mild cognitive impairment with intact stepping responses (MCI: Step), and older adults with mild cognitive impairment with Timber falls (MCI: Timber). Kinematic outcomes included A) fall rate, B) margin of stability (MOS), C) recovery step length (SL), D) step initiation time, and E) step execution time. Boxes are used to indicate the interquartile range (3rd-1st quartile), the horizontal line within the box represents the median, the “X” within the box indicates the mean value, and bars show the minimum and maximum data values (excluding outliers). Data points identified as outliers (> 3rd quartile + 1.5 × interquartile range, or < 1.^st^ quartile – 1.5 × interquartile range) are shown as individual points. Significant group differences are indicated by asterisks (*indicates p < 0.05, **indicates p < 0.01, ***indicates p < 0.001)

There was a significant main effect of group on fall rate (X^2^(3) = 45.147, *p* < *0.001*), MOS (F(3,90) = 13.849, *p* < *0.001*), step initiation time (F(3,90) = 58.964, *p* < *0.001*), and recovery step length (F(3,90) = 13.849, *p* < *0.001*). Fall rate was higher in CIOA, MCI: Step, and MCI: Timber compared to YA (*p* < *0.001*), and higher in MCI: Timber compared to CIOA and MCI: Step (*p* < *0.001*) (Fig. 3A). MOS was lower in CIOA (*p* < *0.001*), MCI: Step (*p* = *0.006*), and MCI: Timber (*p* < *0.001*) compared to YA, and lower in MCI: Timber compared to CIOA (*p* = *0.016*) and MCI: Step (*p* = *0.005*) (Fig. 3B). Recovery step length was shorter in CIOA (*p* = *0.001*), MCI: Step (p = 0.036), and MCI: Timber (*p* < *0.001*) compared to YA, and shorter in MCI: Timber compared to CIOA (*p* = *0.029*) and MCI: Step (*p* = *0.009*) (Fig. 3C). Step initiation time was longer in MCI: Timber compared to YA, CIOA, and MCI: Step (*p’s* < *0.001*), and delayed in MCI: Step compared to YA (*p* = *0.003*) (Fig. 3D). There was no significant main effect of group on step execution time (F(3,89) = 2.186, *p* = *0.095*) (Fig. 3E).

### Muscle onset latencies

**Fig. 4:**
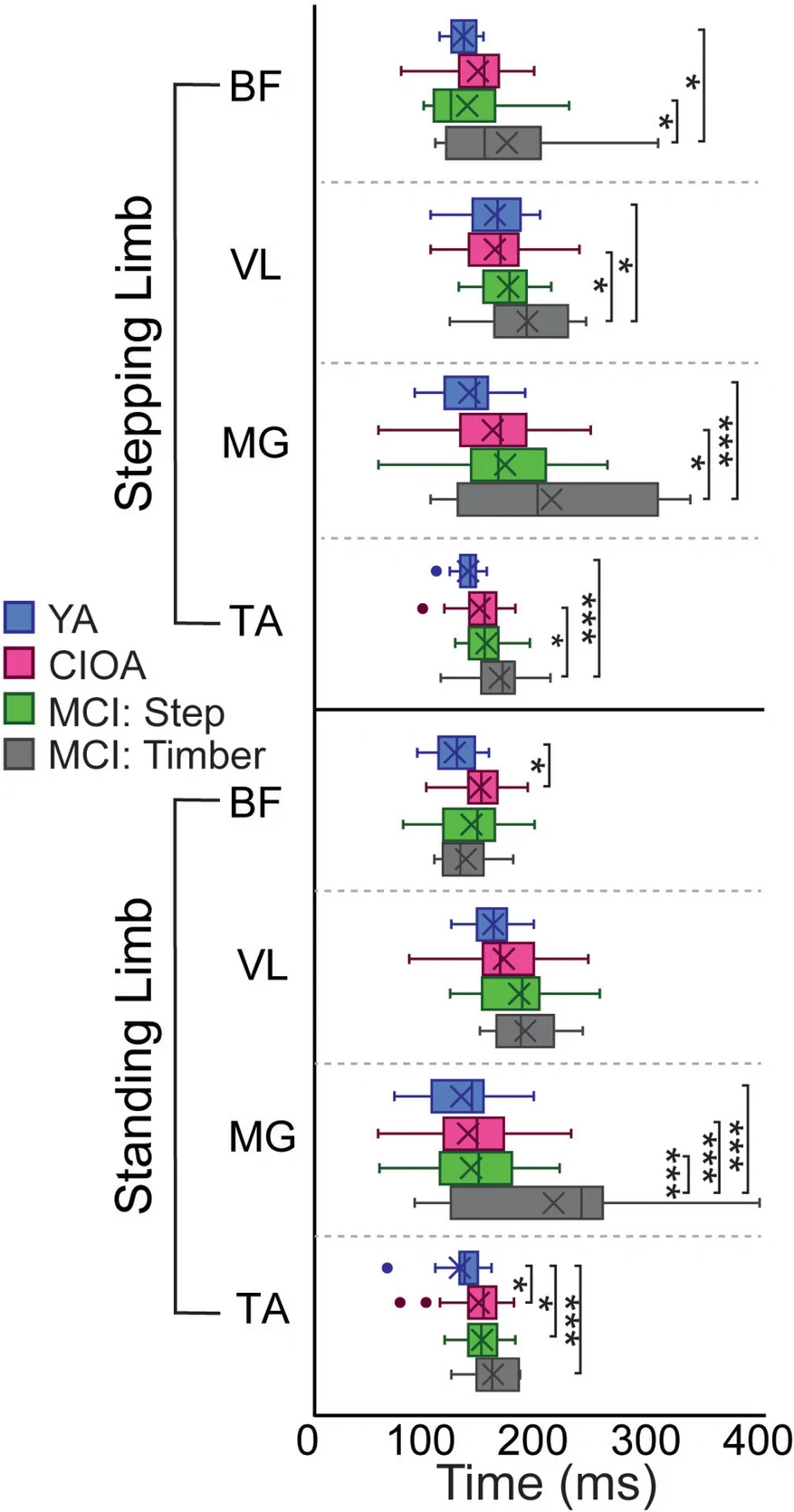
Onset latencies in muscles on the recovery stepping limb and standing limb in young adults (YA), cognitively intact older adults (CIOA), older adults with mild cognitive impairment with intact stepping responses (MCI: Step), and older adults with mild cognitive impairment with Timber falls (MCI: Timber) (groups are pictured in this order from top to bottom for each muscle). Onset latencies were calculated for the biceps femoris (BF), vastus lateralis (VL), medial gastrocnemius (MG), and tibialis anterior (TA), and time 0 indicates perturbation onset. Boxes are used to indicate the interquartile range (3rd-1st quartile), the horizontal line within the box represents the median, the “X” within the box indicates the mean value, and bars show the minimum and maximum data values (excluding outliers). Data points identified as outliers (> 3rd quartile + 1.5 × interquartile range, or < 1.^st^ quartile – 1.5 × interquartile range) are shown as individual points. Significant differences in muscle onset latencies between groups are indicated by asterisks (*indicates p < 0.05; **indicates p < 0.01;***indicates p < 0.001)

On the recovery stepping limb, there were significant main effects of group on the onset latency of the BF (F(3,90) = 3.701, *p* = *0.015*), VL (F(3,90) = 4.074, *p* = *0.009*), MG (F(3,90) = 5.756, *p* = *0.001*), and TA (F(3,90) = 6.484, *p* < *0.001*) (Fig. 4). Stepping limb BF onset was delayed in MCI: Timber compared to YA (*p* = *0.016*) and MCI: Step (*p* = *0.031*). Stepping limb VL, MG, and TA onsets were delayed in MCI: Timber compared to YA and CIOA (*p* ≤ *0.027*).

On the stance limb, there were significant main effects of group on the onset latency of the BF (F(3,90) = 4.589, *p* = *0.005*), VL (F(3,90) = 2.995, *p* = *0.035*), MG (F(3,90) = 8.619, *p* < *0.001*), and TA (F(3,90) = 5.986, *p* < *0.001*) (Fig. 4). Standing limb BF onset was delayed in CIOA compared to YA (*p* = *0.003*). Standing limb MG onset was delayed in MCI: Timber compared to YA, CIOA, and MCI: Step (*p’s* < *0.001*). Standing limb TA onset was delayed in CIOA (*p* = *0.028*), MCI: Step (*p* = *0.015*), and MCI: Timber (*p* < *0.001*) compared to YA. For standing limb VL, there were no significant pairwise comparisons after Bonferroni correction.

### Muscle Synergies

There was a significant group effect on the number of synergies (F(3,90) = 3.809, *p* = *0.013*). MCI: Timber recruited significantly higher number of synergies (4.00 ± 0.71) than YA (3.30 ± 0.47, *p* = *0.020*), CIOA (3.37 ± 0.63, *p* = *0.020*), and MCI: Step (3.35 ± 0.78, *p* = *0.030*).

**Fig. 5:**
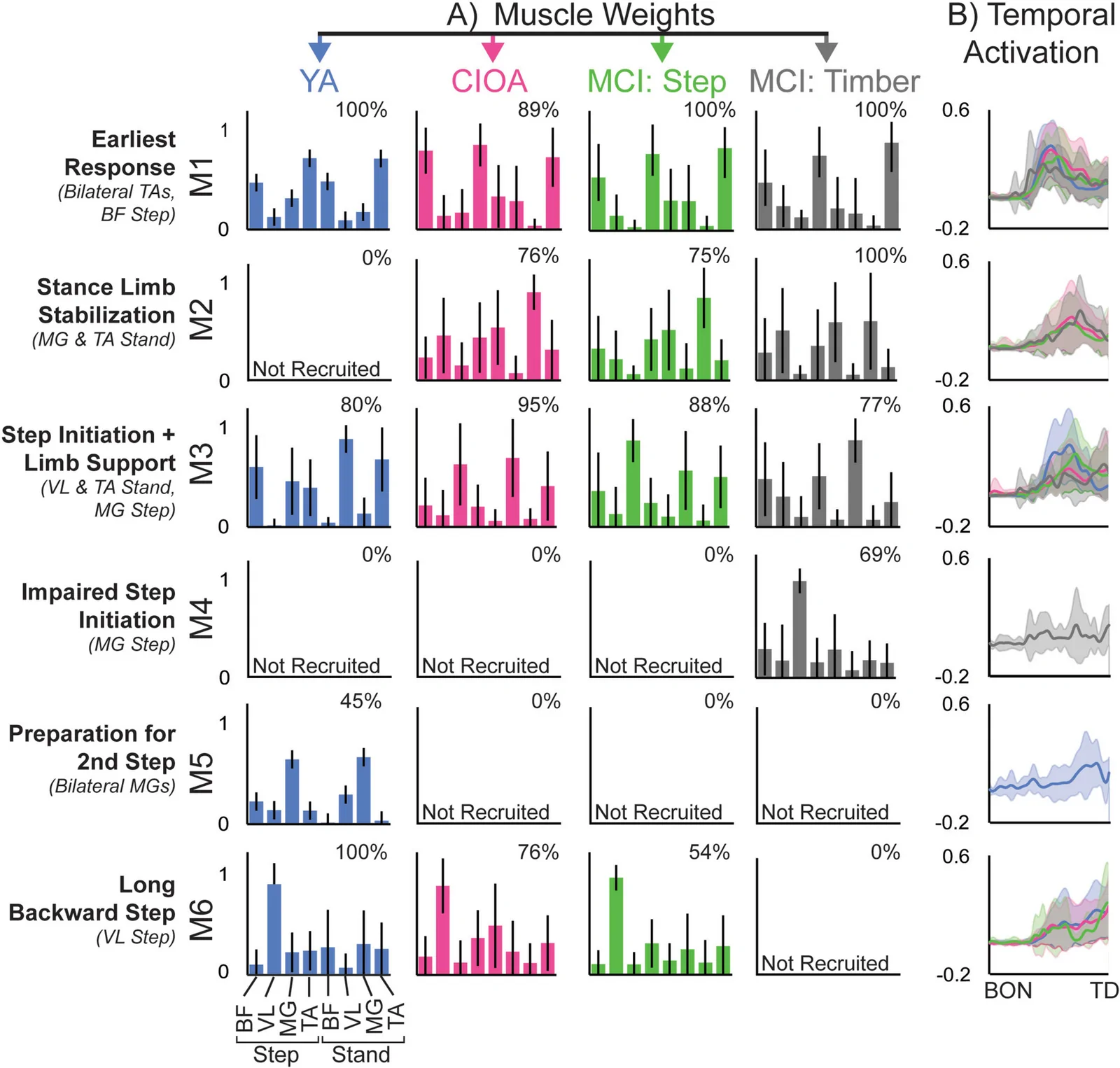
Muscle synergies recruited by young adults (YA), cognitively intact older adults (CIOA), older adults with mild cognitive impairment with intact stepping responses (MCI: Step), and older adults with mild cognitive impairment with Timber responses (MCI: Timber) during unexpected perturbation exposure. Muscle synergies were extracted from electromyography data collected from the biceps femoris (BF), vastus lateralis (VL), medial gastrocnemius (MG), and tibialis anterior (TA) on the recovery stepping limb and standing limb. Cluster analysis revealed six synergies (M1-6), which were ordered by their peak temporal activation after perturbation onset. Each muscle synergy consists of a spatial structure indicated by the contribution/weight of each muscle (A) and is recruited using a specific temporal activation pattern from perturbation onset (BON) to recovery step touchdown (TD) (B). Bar graphs represent the spatial structure of each synergy for each group (mean ± SD contribution of each muscle), and the percentage in the top right corner indicates the percentage of participants within each group who recruited that specific synergy. Synergies which were not recruited by a specific group are labeled as “Not Recruited.” The potential role of each synergy is labeled on the lefthand side, which is a postulation based on which muscles are major contributors (indicated in parentheses) and the order of activation

Cluster analysis revealed six total muscle synergies utilized during reactive balance (M1-6) (Fig. 5). There were two common synergies recruited by all groups (M1, M3), one synergy recruited only by older adult groups (CIOA, MCI: Step, MCI: Timber) (M2), and one synergy recruited only by YA (M5). There was also one synergy recruited only by MCI: Timber (M4), and one synergy which was recruited by all groups except for MCI: Timber (M6). The structural similarity of synergies between groups (i.e., Pearson correlation coefficients) can be found in Table 2 and each synergy is sequentially described below.

**Table 2:** Similarity of muscle synergy structure (M1-6) between groups, indicated by the Pearson’s correlation coefficient (r) between the average structure vectors of synergies. There were four groups compared, including young adults (YA), cognitively intact older adults (CIOA), older adults with mild cognitive impairment with intact stepping responses (MCI: Step), and older adults with mild cognitive impairment with Timber falls (MCI: Timber). Synergies with lower similarity between groups (r < 0.834) are highlighted in bold text. “N/A” indicates that at least one of the groups did not recruit this synergy

	YA vs. CIOA	YA vs. MCI: Step	YA vs. MCI: Timber	CIOA vs. MCI: Step	CIOA vs. MCI: Timber	MCI: Step vs. MCI: Timber
M1	0.844	0.851	0.868	0.939	0.983	0.964
M2	**N/A**	**N/A**	**N/A**	0.921	0.876	**0.803**
M3	**0.796**	**0.696**	**0.691**	0.920	**0.456**	**0.203**
M4	**N/A**	**N/A**	**N/A**	**N/A**	**N/A**	**N/A**
M5	**N/A**	**N/A**	**N/A**	**N/A**	**N/A**	**N/A**
M6	0.862	0.908	**N/A**	0.894	**N/A**	**N/A**

The first synergy after perturbation onset (M1) was recruited by all groups and almost all (> 89%) participants. The spatial structure of M1 was highly similar (r ≥ 0.844) between groups, and the major contributors (load > 0.4) were the bilateral TAs and stepping limb BF.

M2 was not recruited by YA, but was recruited in ~ 75% of CIOA and MCI: Step, and 100% of MCI: Timber. The spatial structure of M2 was similar between CIOA and MCI: Step (r = 0.921), although had lower similarity between MCI: Step and MCI: Timber (r = 0.803). The largest contributors to M2 were the standing MG and TA, and MCI: Timber showed higher contribution of the stepping VL than MCI: Step.

M3 was recruited by all groups, although showed large differences in spatial structure between groups. CIOA and MCI: Step had similar spatial structure of M3 (r = 0.920), although lower similarity with YA (r ≤ 0.696), and even lower similarity with MCI: Timber (r ≤ 0.456). The major contributors to M3 in YA, CIOA, and MCI: Step were the stepping MG, standing VL, and standing TA. YA additionally showed high contribution of the stepping BF. However, the contribution of the stepping MG and standing TA were relatively much lower in MCI: Timber.

M4 and M5 were both unique synergies recruited by only one group. M4 was only recruited by MCI: Timber (69%) and consisted primarily of the stepping MG, with lower contribution from other muscles. M5 was only recruited by 45% of YA consisted primarily of the bilateral MGs.

Lastly, M6 was recruited by YA, CIOA, and MCI: Step, although was more common in YA (100%) than CIOA (76%) and MCI: Step (54%). M6 was not recruited by MCI: Timber. The structural similarity of M6 was highly similar between groups (r ≥ 0.862) and the major contributor was the stepping VL.

## Discussion

This study investigated potential neuromechanistic causes of reactive balance deficits and Timber falls in OAwMCI. As expected, OAwMCI had a higher prevalence of Timber falls (36%) than CIOA (0%) and YA (0%). Compared to groups with intact reactive stepping, OAwMCI with Timber falls had delayed lower limb muscle onset latencies (particularly on the stepping limb) and showed different muscle synergy patterns which mostly reflected altered coordination of the stepping limb plantarflexors (MG) and knee extensors (VL). OAwMCI with Timber falls also recruited a greater number of muscle synergies than other groups. These results suggest that pathology related to MCI might increase the risk of Timber falls by both 1) delaying the initiation/triggering of reactive balance responses, and 2) affecting the selection and execution of motor commands, thus leading to ineffective reactive stepping responses which fail to restore COM stability.

OAwMCI with Timber falls did not activate stepping limb muscles until ~ 170–210 ms after perturbation onset, while older adults with intact stepping activated stepping limb muscles within ~ 130–170 ms. Out of all stepping limb muscles, OAwMCI with Timber falls showed the largest magnitude delays in the MG (~ 50 ms), which might have contributed to the delays in recovery step initiation that we observed in this group (*see kinematic results*), due to later generation of propulsive impulse via ankle plantarflexion. OAwMCI with Timber falls also showed a significant delay in stepping hamstrings (BF) activation which could have further contributed to later initiation of reactive stepping, as knee flexion is also needed to lift the stepping foot off the ground. OAwMCI with Timber falls additionally showed ~ 70 ms delay in onset of the MG on the standing limb, which could be involved in stabilizing this limb while preparing to initiate reactive stepping.

These delayed muscle onset latencies indicate that MCI-related pathology likely interferes with the initiation/triggering of reactive balance responses. When the CNS perceives that a perturbation has occurred, reactive balance responses are postulated to be triggered from the brainstem [57], with reactive stepping initiated once the COM is perceived to cross a velocity-dependent threshold [58]. The brainstem receives and integrates multiple sensory inputs (e.g., proprioceptive, somatosensory, vestibular, visual) [59, 60], although perturbation detection is proposed to rely primarily on proprioceptive and somatosensory information (e.g., changes in ankle joint position) due to the rapid latency of these systems [61]. OAwMCI are known to have sensorimotor processing and integration deficits (especially involving somatosensory information) [14–16], which could lead to delays in detecting when a perturbation has occurred and/or assessing current COM state. Further, OAwMCI may have lower efficiency of brainstem neural circuits involved in response initiation due to pathological reductions in brainstem grey matter volume [18, 62] and/or slowing of neural transmission due to myelin breakdown [63, 64]. Additionally, it is possible that response triggering in OAwMCI is affected by dysfunction of other sensory systems with afferent projections to the brainstem (e.g., vestibular system). Vestibular dysfunction is disproportionately prevalent in OAwMCI compared to CIOA [65, 66], and multiple studies show that vestibular dysfunction can impair reactive balance control [67–69]. Further, there is initial evidence that vestibular stimulation can increase the sensitivity of the somatosensory system [70], and thus possibly contribute to perturbation detection/response triggering. However, the role of the vestibular system during reactive balance control in OAwMCI is yet to be fully examined.

Our results additionally showed that OAwMCI with Timber falls had different muscle synergy patterns compared to older adults with intact reactive stepping, especially related to coordination of muscles on the stepping limb. For example, M3 mainly consisted of contribution from the stepping MG along with some standing limb muscles (VL, TA) in groups with intact stepping, although OAwMCI with Timber falls had very low contribution of the stepping MG to this synergy, resulting in the similarity being quite low (*r* = *0.203–0.696*). OAwMCI with Timber falls instead recruited a unique synergy which consisted of high contribution from stepping limb MG, although little contribution from standing limb muscles (M4). This might indicate that adults with intact reactive stepping utilize more intricate interlimb coordination during reactive stepping, thus helping to push the stepping foot off the ground while simultaneously providing contralateral limb support (via generation of net extensor torque) to prevent limb collapse [22, 23]. Further, all groups except for OAwMCI with Timber falls recruited M6, which consisted highly of the stepping VL and had a peak activation in the later swing phase of reactive stepping. It is possible that this synergy was related to taking a long backward step (via knee extension), as we observed that OAwMCI with Timber falls had significantly shorter recovery step length than other groups.

These structural differences in recruited muscle synergies indicate that MCI-related pathology may also impair the selection or execution of appropriate motor commands for reactive stepping. Depending on the type of motor action, it is suggested that the structure of muscle synergies are likely encoded at the level of the spinal cord (locomotor tasks) or brainstem (postural tasks) [30, 71], thus creating a pre-existing library of synergies which can be selected from based on the demands of the current task [30, 72]. Specifically, some studies have shown that the structure of muscle synergies remain intact even when cortical input is damaged – for example, in decerebrate cats [73] or in adults with cortical stroke [74]; thus supporting the postulation that the cortex may not be involved in the initial triggering of specific muscle synergy structures. Further, some authors postulate that the rapid latency of reactive muscle activity might not be sufficient to account for a transcortical loop [57, 75]. Thus, the effects of MCI pathology (e.g., sensorimotor processing deficits, reduced efficiency of brainstem neural loops) might not only delay the reactive activation of lower limb muscles (*as indicated by delayed onset latencies*), but additionally affect the structure of the triggered motor commands. One possible explanation is that OAwMCI might not be able to determine which motor commands will be most effective for regaining stability because they cannot effectively/rapidly integrate sensorimotor information regarding the current postural and environmental state or perturbation characteristics (e.g., direction, magnitude). Further, the higher number of synergies that we observed in OAwMCI with Timber falls could have been a compensatory mechanism of ‘over-recruiting’ synergies based on an inability to determine the most optimal combination of synergies.

Once a specific muscle synergy is triggered, MCI pathology may also affect its temporal recruitment (i.e., timing and amplitude of activation) during the execution of reactive stepping, although we did not formally analyze this. The cortex is proposed to become more and more involved in reactive balance control as the response progresses [57], and is likely involved in modulating the temporal recruitment of muscle synergies via projections to descending motor pathways (e.g., corticospinal tract, frontopontine tract) [56, 72, 76]. OAwMCI have shown reduced white matter integrity in these pathways compared to CIOA [77, 78], which could impair the efferent transmission of motor signals, and has been associated with lower reactive COM stability [79]. Additionally, OAwMCI show altered structure/function of the motor cortex and cerebellum (e.g., reduced grey matter volume, decreased functional connectivity between these areas) [17, 19, 34, 80], which could also affect the modulation/scaling of muscle synergies. Further, it is possible that the cortex also indirectly contributes to the activation patterns of muscle synergies via central set, which is updated based on multiple factors (e.g., prior perturbation exposures, changes in sensorimotor conditions) and can allow the CNS to proactively prepare for upcoming perturbations, especially those which are anticipated [81, 82]. Central set could also possibly affect synergy structure by ‘priming’ specific muscle synergies within the brainstem before the perturbation has even occurred [57].

We also observed some age-related differences in the neuromuscular control of reactive balance (both onset latencies and muscle synergies) between younger and older adults, even though this was not the main focus of this study. All older adult groups showed slight delays in onset of the standing TA compared to YA (~ 15–30 ms), and CIOA showed ~ 20 ms delay in onset of the standing BF compared to YA, which could indicate a delay in triggering the initial response to counteract the forward displacement of the BOS as the treadmill accelerated. These results are in line with previous studies which show slight age-related delays in muscle onset latencies during reactive balance tasks [83–85], and could be attributed to well-established age-related declines in sensorimotor function (e.g., proprioception) and CNS processing speed [86, 87]. We also observed some differences in the structure of muscle synergies between younger and older adults. YA did not recruit M2, which mostly consisted of the standing MG and BF, and was possibly a protective strategy used only by older adults which included stiffening the standing limb. Further, only YA recruited M5, which consisted mainly of bilateral MG activity and showed peak activation right before recovery touchdown, which possibly indicated early preparation for initiation of a second recovery step. These neuromuscular differences could have led to less effective perturbation responses, thus contributing to the higher fall rate and lower reactive COM stability that we observed in older adult groups. It should also be noted that we found a slightly higher mean age in MCI: Timber compared to MCI: Step (even though the group difference was not significant). However, we found that including age as a covariate in preliminary analyses did not affect the reported results, suggesting that Timber falls in OAwMCI were not simply due to age-related changes. We did not include age as a covariate in our final analyses, as we also wanted to explore age-related changes between young adults and older adult groups.

These mechanistic results do provide some clinical applications, especially related to the design of future fall prevention interventions for OAwMCI. Most existing fall prevention interventions for OAwMCI have only involved volitional-based exercises [88–90], and their effects on improving reactive balance control have not been studied. Task-specific interventions which train reactive balance control mechanisms (e.g., perturbation-based training) could be more effective for implicitly training reactive stepping in OAwMCI, as they have shown robust benefits for reducing laboratory fall rate and improving reactive COM stability in CIOA [91–93], potentially due to underlying improvements in neuromuscular coordination [53]. However, reactive balance training paradigms might need to be tailored to MCI-specific deficits to most effectively improve both the initiation and execution of reactive stepping. For example, improving sensorimotor processing and perception may enhance perturbation detection in OAwMCI, and perturbation-based training protocols may consider supplemental exercises which challenge the proprioceptive system (e.g., balancing on an unstable/uneven surface) [94, 95] or exercises which target multi-sensory integration (e.g., exergaming) [96, 97]. Likewise, perturbation-based training protocols could be tailored to improve the execution of reactive stepping in OAwMCI by including additional agility stepping exercises or providing biofeedback regarding stepping parameters (e.g., length, timing). Additionally, incorporating dual task conditions into perturbation-based training could mimic real-life conditions and target reactive stepping while also simultaneously improving attentional control/cognitive processing [98, 99].

There are some limitations of this study which we acknowledge. First, we assessed neuromuscular responses to unidirectional perturbations delivered during stance, while many perturbations in daily living occur in multiple directions or while walking [100–102]. Therefore, there may be limited generalizability of our results to the wide variety of perturbations which occur in daily living (e.g., slips, trips, lateral perturbations), and future studies would be warranted to examine how OAwMCI respond to multi-directional perturbations which require different recovery strategies (e.g., backward recovery stepping for slip-like perturbations vs. forward recovery stepping for trip-like perturbations). Additionally, we only explored the effects of cognitive impairment on responses to a singular perturbation exposure in order to avoid the effects of motor adaptation on reactive balance responses, which may occur at a different rate in OAwMCI due to motor learning deficits [103, 104]. Thus, future studies could also examine neuromuscular adaptation to repeated perturbations in OAwMCI vs. CIOA. We also only collected EMG data from lower limb muscles which were primarily involved in implementing a backwards recovery step in the sagittal plane (i.e., hip and knee flexion/extension, ankle plantarflexion/dorsiflexion). However, other muscles can still contribute to the reactive balance response (e.g., trunk muscles, lower limb abductors/adductors) and recording EMG data from a larger set of muscles may allow for even more in-depth analysis of how cognitive impairment affects muscle synergies during reactive balance control. Lastly, it should be considered that we normalized EMG signals to the maximum activation within the response window, although there are alternative methods of EMG normalization which could also be considered during analysis (e.g., maximal isometric voluntary contraction (MVIC) method). However, one study found that extracted muscle synergy patterns were highly similar between EMG data normalized by the maximum in-task signal compared to EMG data normalized by MVICs [105], suggesting that the normalization method may have had a minimal effect on study results.

In conclusion, we found that pathology related to MCI can affect the neuromuscular control of reactive balance and might increase the prevalence of Timber falls due to both delayed initiation and impaired execution of reactive stepping. Specifically, OAwMCI with Timber falls had delayed initiation of corrective muscle activity, which could possibly be due to impaired sensorimotor processing and integration, as well as structural damage in motor areas responsible for response initiation (i.e., brainstem). Further, OAwMCI with Timber falls recruited more synergies than those with intact stepping and had altered muscle synergy patterns, which could be attributed to impaired selection of optimal motor commands and/or damage in descending motor pathways (e.g., corticospinal tract). Fall prevention interventions for OAwMCI may consider ways to improve sensorimotor perception/processing and effective reactive stepping mechanisms in this population. Further, future studies could use neuroimaging to investigate subcortical and cortical correlates of Timber falls in OAwMCI.

## Data Availability

The study data will be made available from the corresponding author upon reasonable request.
